# Exploring Spinal Subarachnoid Hemorrhage: A Neurosurgical Case Series

**DOI:** 10.7759/cureus.45627

**Published:** 2023-09-20

**Authors:** Kiran Sankarappan, Dakota Doucet, Samuel R Daly, Anthony V Nguyen, David Garrett, Walter S Lesley, Dongxia Feng, Awais Z Vance, Jason H Huang

**Affiliations:** 1 Neurosurgery, Baylor Scott & White Medical Center, Temple, USA

**Keywords:** intraoperative neurologic monitoring, spinal subarachnoid cyst, neurologic exam, spinal decompression, dural arteriovenous fistula (davf), spinal angiogram, spinal subarachnoid hemorrhage

## Abstract

Spinal subarachnoid hemorrhage (SSAH) is a rare condition that can cause spinal cord or nerve root compression and permanent neurologic damage. The reported etiologies include trauma, vascular malformations or aneurysms, coagulopathies, neoplasms, autoimmune disease, and spontaneous hemorrhage. If there is evidence of neurologic deterioration, it is commonly managed as a surgical emergency, but cases of conservative management have also been reported. In this case series, we present three patients who suffered from SSAH. The first was a spontaneous cervical SSAH that occurred following cardiac catheterization, the second was a spontaneous thoracolumbar SSAH in a patient with a known history of coagulopathy, and the third was a thoracolumbar SSAH that was caused by a dural arteriovenous fistula (dAVF). All three patients exhibited neurologic deficits and thus underwent emergent decompression and hematoma evacuation. The patient with the dAVF also required open ligation of the fistula. Following surgical intervention, all three patients regained at least partial neurologic function, but one patient developed symptomatic arachnoid cysts that required further intervention. The presented case series highlights the importance and time-sensitivity of surgical decompression in patients experiencing neurologic deficits from SSAH. These cases underscore the urgency of timely neurosurgical intervention to mitigate neurologic impairment and add insights to the existing literature on this rare condition.

## Introduction

Spinal subarachnoid hemorrhage (SSAH) is a rare but serious disease with a multitude of etiologies. A large review article and meta-analysis published in 2003 reported only 96 cases of SSAH between 1826 and 1996, displaying the disease's historical rarity [1]. The patients were between three and 83 years old (peak incidence between 15-20 years old), and there was a 2:1 male-to-female predominance. The differential diagnosis for SSAH includes neoplasms, vascular pathologies, coagulopathy, anticoagulation therapy, autoimmune diseases, connective tissue diseases, trauma/iatrogenic, pregnancy-related, and idiopathic hemorrhage [1,2]. Most of the primary literature on SSAH is single case reports or small case series, so accurate epidemiologic data, the most effective treatment strategies, and the outcomes of SSAH are largely unknown.

The clinical presentation of SSAH is usually a neurologic deficit associated with spinal cord or nerve root compression that varies depending on the level of the lesion and associated pathology. Most patients that present with symptoms of mass effect on spinal neural elements present acutely with a complete or incomplete spinal injury, and less than 15% present in a subacute or chronic manner [1]. Urinary and bowel symptoms tend to be more common in those who present with an acute symptom onset. In addition to symptoms of spinal cord and nerve root compression, up to a third of patients can present with symptoms of cerebral subarachnoid hemorrhage, such as headaches, neck pain, disturbances of consciousness, and seizures [1]. These symptoms are likely caused by meningeal irritation and extension of the hemorrhage throughout the subarachnoid space. Regardless of the presenting symptoms, advanced imaging is necessary for a comprehensive understanding of the pathology. Computed tomography (CT) with myelography can characterize a filling defect referred to as "capping" in SSAH; however, CT myelography fails to provide a precise picture due to the inability of contrast to permeate the plane between the spinal cord and the hematoma [2,3]. Magnetic resonance imaging (MRI) with and without contrast is the preferred diagnostic modality given its resolution and ability to detect blood products as well as underlying enhancing etiologies. If vascular etiologies are suspected, CT angiography or spinal angiograms can be used to attempt to establish the underlying cause.

SSAH is typically managed as a surgical emergency if there are signs of acute neurologic deterioration, and patient recovery hinges upon preoperative clinical status as well as early intervention [1,2,4-6]. There have also been reports of successful conservative management in patients without neurologic dysfunction [7,8]. Overall, there is a paucity of evidence assessing the optimal timing of surgery, the necessity of endovascular evaluation, and whether neurosurgical decompression and/or hematoma evacuation is necessary in the absence of neurologic deficits. The cases summarized here provide valuable information to the literature on this rare condition.

## Case presentation

Case one

**Figure 1:**
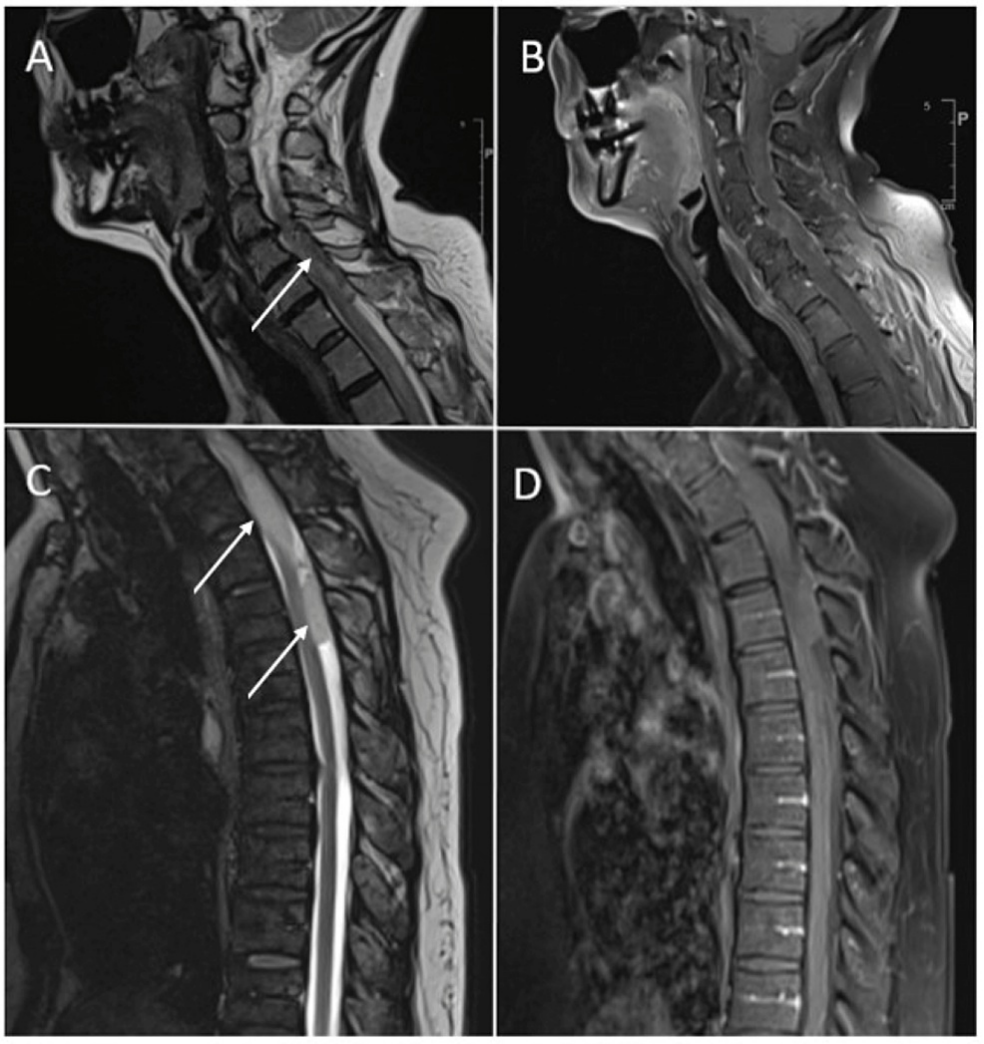
Sagittal MRI images from case one (A) T2 sequence of the patient's cervical spine showing intradural hematoma (white arrow); (B) Post-contrast T1 sequence of the patient's cervical spine showing no abnormal enhancement; (C) T2 sequence of the patient's thoracic spine showing the intradural hematoma (white arrows); (D) Post-contrast T1 sequence of the patient's thoracic spine showing no abnormal enhancement.

**Figure 2:**
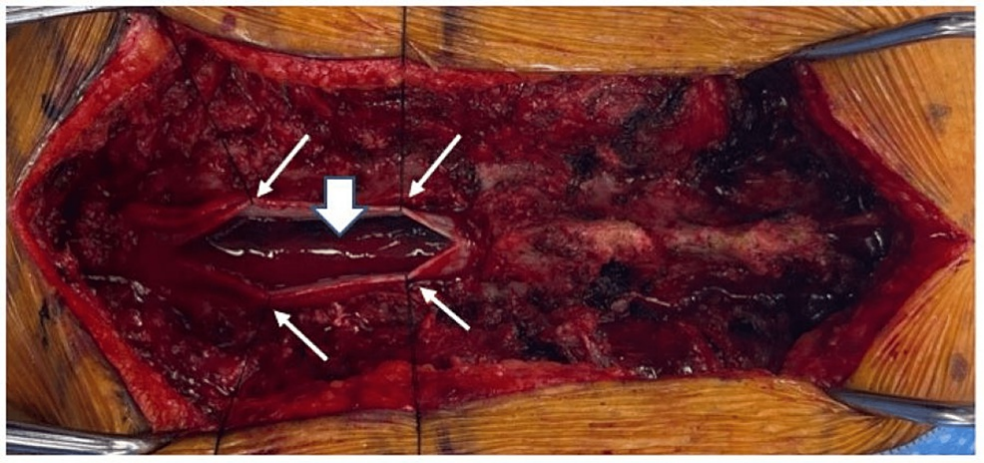
Intraoperative image of case one The dura has been opened at the midline and tacked up with sutures (small white arrows), and the subarachnoid hematoma is visible (large white arrow).

A 60-year-old otherwise healthy female who had three weeks of syncope with exertion and lightheadedness when extending her neck underwent an elective heart catheterization as part of her syncopal work-up. The procedure was uneventful, aside from coronary artery spasm. Two hours after waking from anesthesia, she reported an acute onset of severe headache that radiated into her neck and bilateral shoulders. At that time, her headache was associated with weakness in her right arm and bilateral lower extremities, as well as hypoesthesia and paresthesia in her entire body below her chest. Non-contrast CT head and CT angiography (CTA) head/neck did not show any evidence of intracranial hemorrhage or vascular abnormality. The next day, the hypoesthesia and paresthesia had ascended to affect her arms, and she was found to be incontinent. Contrast-enhanced MRI brain, cervical spine, and thoracic spine demonstrated non-enhancing, lobulated, intradural extramedullary lesions, which were consistent with intradural hematomas (Figure 1). She was immediately evaluated by the neurosurgical service. On neurologic exam, she was alert and fully oriented, she had a C4 sensory level with right-sided allodynia, and she had objective weakness in her right upper extremity as well as her bilateral lower extremities. She underwent emergent C5-T3 laminectomies and intradural exploration. After the dura was opened, a hematoma was noted to be confined within the subarachnoid space (Figure 2). This was evacuated until cerebrospinal fluid began to appear at the cranial edge of the surgical field. The dura was repaired by primary closure, and she was transferred to the intensive care unit. At the time of discharge to inpatient rehabilitation, approximately two weeks later, her strength exam had improved. Her right upper extremity strength was nearly 5/5 in all muscle groups, and her bilateral lower extremity strength had partially improved, although she was not yet antigravity in the proximal muscle groups.

Two months postoperatively, she was taken back to the operating room for a catheter cerebrospinal angiogram, which did not show any vascular pathology. Follow-up MRIs with and without contrast were done three months postoperatively, which showed myelomalacia in her cervical spinal cord extending from C5 through T1 without any target for surgical decompression. At that time, the strength in her left leg was 4/5, and the strength in her right leg was 2/5. 

Case two

**Figure 3:**
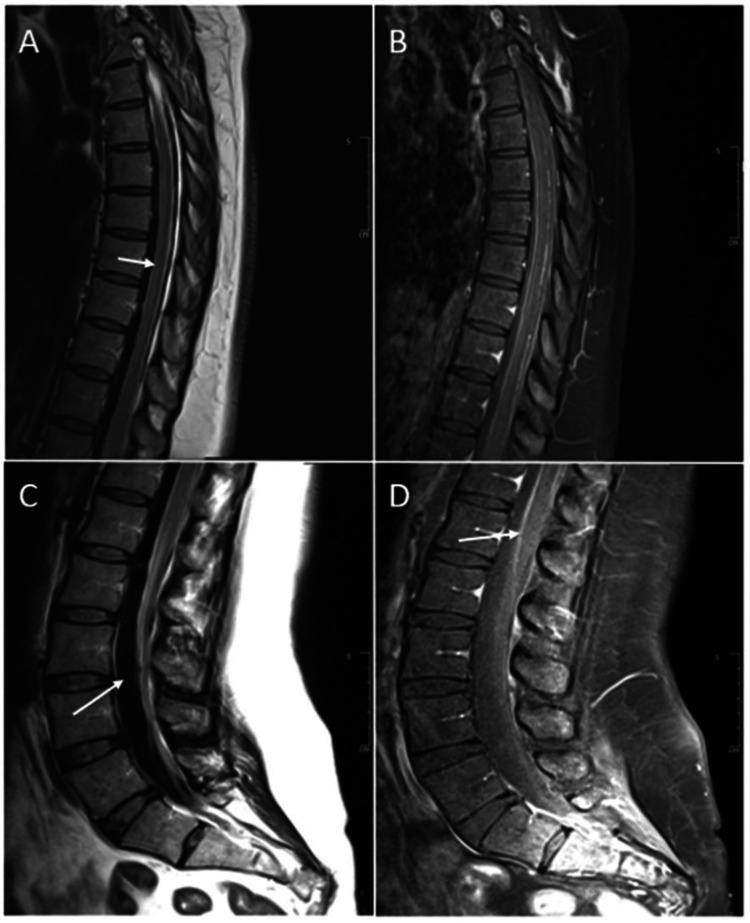
Sagittal MRI images from case two (A) T2 sequence of the patient's thoracic spine showing intradural hematoma (white arrow); (B) Post-contrast T1 sequence of the patient's thoracic spine showing leptomeningeal enhancement presumably due to irritation of the thoracic cord and nerve roots from the hemorrhage; (C) T2 sequence of the patient's lumbar spine showing intradural hematoma (white arrow); (D) Post-contrast T1 sequence of the patient's lumbar spine showing leptomeningeal enhancement around the conus (white arrow).

**Figure 4:**
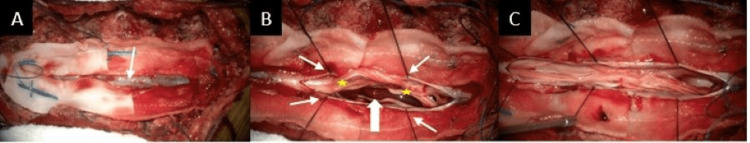
Intraoperative images of case two hematoma evacuation (A) The thecal sac appears to be bulging posteriorly (white arrow); (B) The dura has been opened at the midline and tacked up with sutures (small white arrows), and both the nerve roots (yellow stars), as well as the subarachnoid hematoma (large white arrow), are visible; (C) The hematoma has been evacuated.

A 45-year-old female with a history of hypertension, as well as multiple deep vein thromboses (DVTs) and pulmonary embolisms (PEs) that she had previously been taking warfarin for, presented to the emergency department with several days of abdominal pain, nausea/vomiting, and diarrhea. She was admitted to the hospital with pneumonia and possible myocardial infarction, so she was given aspirin (325 mg) and started on therapeutic anticoagulation. On the evening of her admission, she reported that her legs had been weak for four days, and she had been experiencing urinary retention and incontinence for two days. On neurologic exam, she had a thoracolumbar sensory level (T10 on the left, and L1 on the right) and objective weakness in her bilateral lower extremities (bilateral hip flexion 3/5 and distal muscle groups 2/5 bilaterally). She had normal reflexes in her bilateral lower extremities with no clonus, and her rectal tone was intact. CT of her lumbar spine did not show any acute pathology, but follow-up MRIs were done given her neurologic exam, and it revealed an intradural hematoma that spanned from the mid-thoracic region through her entire lumbar spine (Figure 3). Her anticoagulation and aspirin were both reversed, and she was taken emergently to the operating room for L2-L5 laminectomies, intradural exploration, and evacuation of the subarachnoid hematoma (Figure 4). The subarachnoid space was irrigated, and the dura was closed primarily. Her urinary retention persisted, but her bilateral lower extremity strength improved to 5/5 by postoperative day one. The Hematology service was consulted and completed a broad work-up for coagulopathy, which was ultimately negative.

Two weeks postoperatively, the patient was admitted to the hospital with worsening back pain, right leg radiculopathy, and 4/5 strength in her proximal right lower extremity. MRI of her lumbar spine revealed partial re-accumulation of blood in the subarachnoid space without significant nerve root compression. A cerebrospinal catheter angiogram was done, which was negative for any vascular pathology. Her lower extremity strength returned to 5/5 with a modified regimen of pain medication, and she was discharged from the hospital.

**Figure 5:**
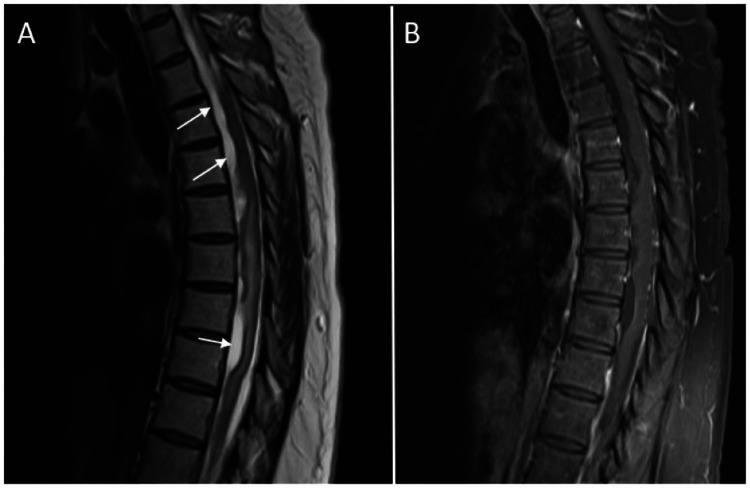
Postoperative sagittal MRI images from case two Sagittal MRI images of the patient's thoracic spine from case two at three months postoperatively showed arachnoid cysts. (A) T2 sequence showing the arachnoid cysts (white arrows); (B) Post-contrast T1 sequence showing no abnormal enhancement.

**Figure 6:**
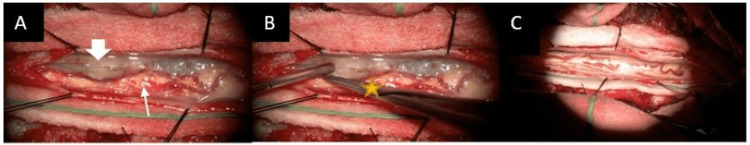
Intraoperative image of case two cyst fenestration (A) The dura has been opened at the midline and tacked up with sutures. The spinal cord (small white arrow) is being displaced by the arachnoid cyst (large white arrow); (B) The cyst is being fenestrated with an arachnoid knife (yellow star); (C) Following cyst fenestration, the mass effect on the spinal has been relieved.

Routine follow-up MRIs of her spine were done three months postoperatively, which showed interval development of multiple subarachnoid fluid collections and displacement of the thoracic spinal cord (Figure 5). She was seen in the clinic shortly after the MRI was done, and her exam was notable for 4/5 strength throughout her right leg and 3+ patellar/Achilles reflexes bilaterally. Given these findings, she was taken to the operating room for T6-T8 laminectomies and intradural exploration. Upon opening the dura, arachnoid cysts were found to be displacing the thoracic spinal cord anterolaterally (Figure 6). The cysts were fenestrated along the length of the durotomy using micro-scissors and an arachnoid knife, which resulted in the spinal cord returning to midline and re-expanding. On postoperative day one, the patient's strength had returned to 5/5 throughout both of her lower extremities. 

Case three 

**Figure 7:**
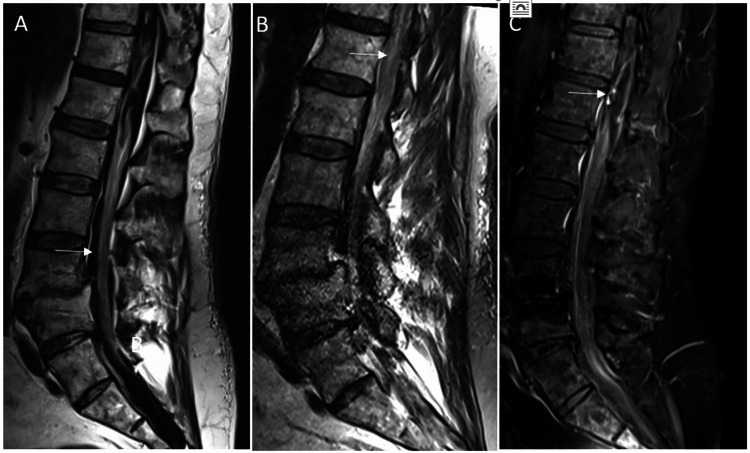
Sagittal MRI images from case three (A) T2 sequence showing the intradural hematoma (white arrow); (B) T2 sequence showing hyperintensity at the conus, consistent with a possible infarct (white arrow); (C) T1 post-contrast sequence showing abnormal enhancement in the ventral spinal canal (white arrow).

A 65-year-old man with a past medical history including hypertension, diabetes, and coronary artery disease/myocardial infarction (on aspirin 325 mg per day) presented to an outside facility with three days of progressively worsening severe low back pain to the point that he was unable to walk. A CT of his lumbar spine was done, which was concerning for epidural hematoma, so he was transferred to our institution for a higher level of care. On arrival here, he also reported new onset urinary retention. On neurologic exam, his strength was severely pain-limited but near full strength in his left lower extremity and right ankle. Patellar and ankle reflexes were 3+ bilaterally, and his sensation was intact. MRIs of his thoracic and lumbar spine were done on arrival at our institution, which showed an intradural extramedullary hematoma, possible infarct at the conus given T2 hyperintense signal, and abnormal enhancement surrounding the spinal cord near T11 concerning for a dural arteriovenous fistula (dAVF, Figure 7).

**Figure 8:**
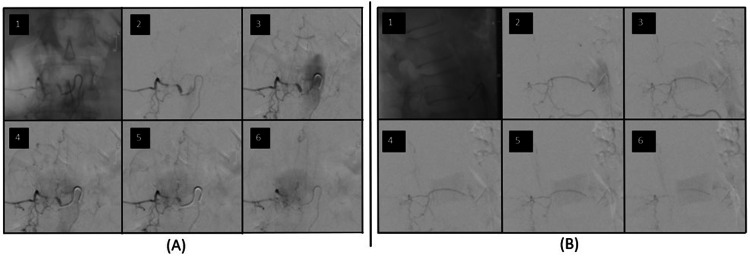
Spinal angiogram from case three Sequential images from the spinal angiogram of a right L1 radicular branch. The nidus of the dural AVF is fed from a linear ascending artery off the right L1 radicular branch and drains into veins on the posterior aspect of the spinal cord. (A) Anterior/posterior view; (B) Lateral view.

He was taken emergently for a spinal angiogram, confirming a spinal dAVF nidus at T11-12 that was supplied by a right L1 radicular artery (Figure 8). The feeding nidus was not able to be accessed with a microcatheter, so embolization of the dAVF was not possible. The patient was thus taken to the operating room immediately following the angiogram for spinal decompression and open ligation of the fistula. Decompression was achieved with T11-L1 laminectomies and midline durotomy for evacuation of the subarachnoid hematoma. Once the hematoma was evacuated, prominent veins were seen extending from the right T11, T12, and L1 nerve root sleeves intradurally. Indocyanine green (ICG) angiography suggested that these veins were filling early, so sequential temporary clips were placed on each vein without any changes in somatosensory evoked potentials (SSEPs), motor evoked potentials (MEPs), or electromyography (EMG). These veins were then coagulated and cut. A feeding artery and vein were seen on either side of a round clot near T12 that appeared to be either a pial AVF or aneurysm. The lesion was gently and circumferentially dissected out, and temporary clips were placed on the artery and vein. There were no changes in SSEPs, MEPs, or EMG, so the vessels were coagulated and clipped. A second ICG angiogram did not show any early filling veins. The dura was closed primarily. 

On postoperative day one, the patient remained at full strength in his left lower extremity and right ankle, and his right hip flexion strength was pain-limited but was at least 3/5. His pain level steadily improved, and by discharge on postoperative day ten, he was able to walk about ten steps with assistance. He was seen in the clinic two months postoperatively, and with the exception of right anterior thigh numbness, he had regained all neurologic function. A follow-up spinal angiogram was done eight months postoperatively, which did not demonstrate any residual or recurrent dAVF. 

## Discussion

In this article, we present three cases of SSAH at various spinal levels and with various etiologies. In the first case, a 60-year-old otherwise healthy female underwent an elective cardiac catheterization to rule out cardiogenic syncope and developed a headache and acute neurologic deficits perioperatively. This case represents a rare location of SSAH extending to the upper cervical spine [1,2], as well as an unconventional presenting symptom (headache), which was likely due to cranial meningeal irritation. Given that a follow-up spinal angiogram and follow-up MRI's were negative, there is no definitive identifiable etiology for her hemorrhage, suggesting that it may have been spontaneous, or it could have been related to anticoagulation administered during cardiac catheterization. This case also highlights the potential for neurologic recovery with early recognition and early surgical evacuation in the setting of rapid symptom progression [1,2,4,5,9].

In the second case, we presented a 45-year-old female that developed a SSAH in the setting of anti-platelet treatment, anticoagulation, and a potential underlying coagulopathy. She exhibited remarkable recovery following the initial treatment, but she later developed multiple clinically significant subarachnoid cysts (SAC) throughout her thoracic spine. SAC was first observed in 1943 in a patient that developed lower extremity weakness and had a history of cerebral subarachnoid hemorrhage as a result of an underlying coagulopathy [10]. SACs are postulated to develop from an inflammatory reaction within the leptomeninges to blood products in the cerebrospinal fluid (CSF), but the precise mechanism may be more complex and multifactorial [11,12]. In a case reported by Ginanneschi, et al., a previously healthy 57-year-old female developed a thoracic SAC 10 months after SSAH [13]. While multiple treatments were attempted over the course of eight years, the patient continued to experience worsening symptoms. Velz, et al. reported a case of a 51-year-old female with a spinal SAH at T5-7, who underwent conservative treatment [10]. Three months later, the patient was presented with new neurologic symptoms which were attributed to septated arachnoid cysts that spanned from T4 to T7. The patient underwent marsupialization of the cysts with placement of a shunt, but the cyst recurred. Avoiding recurrence of spinal SAC is challenging, and the most effective treatment option is not well known. Fenestration of the largest cyst was chosen in the case presented here due to the extent of the cyst burden along the patients' entire thoracic spine, but shunting is also an option for localized SACs [14].

The third case presented in this article of a 65-year-old male on full dose aspirin for a history of myocardial infarction is an example of a vascular malformation (dAVF) leading to SSAH. Dural AVF is one of the more common spinal vascular malformations, but other vascular causes include arteriovenous malformations, spinal aneurysms, coarctation of the aorta, and Moyamoya disease [2,15]. Spinal angiography was able to confirm the diagnosis, and endovascular treatment can be effective [16], but it is worth noting that in a retrospective sample of 46 patients with a spinal dAVF, 15% had an initial angiogram that was negative [17]. Surgical evacuation of the hematoma and ligation of the dAVF can lead to favorable outcomes, but the ultimate prognosis also depends on factors such as preoperative spinal cord dysfunction, patient age, duration of symptoms, and the severity of the hemorrhage [5,18-20].

This case series highlights the diversity in presentation, etiologies, and outcomes of SSAH. All three cases have unique clinical aspects and possible associated risk factors for SSAH. We acknowledge that our case series has a limited follow-up interval of the patients, and further research is necessary to determine long-term outcomes of various treatment modalities.

## Conclusions

SSAH is a rare pathology with a wide range of etiologies, including vascular malformations, tumor-associated hemorrhages, trauma, and underlying systemic disease. While the diagnosis of an intradural hematoma has become more accessible due to the widespread availability of advanced imaging, determining the underlying cause and best treatment remains a challenging clinical scenario for clinicians. In this case series, we highlight the diagnostic challenges, the various possible etiologies, and the outcomes of early surgical evacuation of the hematoma in the setting of neurologic compromise. We also report a potential long-term complication of the inflammatory response associated with subarachnoid blood - the formation of clinically significant arachnoid cysts. Given the limited literature available on SSAH, this case series assumes clinical importance as it contributes valuable insights to the existing body of knowledge.
